# Reassessing shelter dogs’ use of human communicative cues in the standard object-choice task

**DOI:** 10.1371/journal.pone.0213166

**Published:** 2019-03-07

**Authors:** Tara Osborne, Nicholas John Mulcahy

**Affiliations:** School of Psychology, McElwain Building, University of Queensland, St Lucia, Brisbane, Queensland, Australia; Consiglio Nazionale delle Ricerche, ITALY

## Abstract

Unlike other animal species, domesticated pet dogs reliably use a range of human communicative cues to find a hidden reward in the object-choice task. One explanation for this finding is that dogs evolved skills for understanding human communicative behaviour during and as a result of human domestication. However, contrary to this domestication hypothesis, Udell et al. found domesticated shelter dogs failed to locate a hidden reward using a human’s distal point cue, a cue pet dogs easily use. Hare et al., however, suggested the unorthodox methods used in Udell et al.’s object-choice task resulted in the shelter dogs failing to use human cues. In support of this, Hare et al. found that shelter dogs could use a human communicative pointing cue when tested with a standard object-choice task method. Yet in contrast to Udell et al., Hare at al. used a much simpler proximal cue that cannot exclude success based on stimulus enhancement rather than an understanding of the cue’s communicative nature. We therefore addressed this issue by testing shelter dogs’ abilities to use a range of proximal and distal human communicative cues in a standard object-choice task. We found shelter dogs could use proximal cues that may involve stimulus enhancement, but they continuously failed to use distal cues that excluded this possibility. Object-choice tasks with dogs typically involve non-vocalised human cues. We tested if vocalising would help shelter dogs to use distal cues. We found shelter dogs could use a vocalised distal continuous cue when the subject’s name was called during cue presentation. It is therefore possible that vocalised cues help domesticated dogs learn about non-vocalised human communicative cues. Overall our results do not support that domesticated dogs’ understanding of human communicative cues is a direct result of the domestication process.

## Introduction

It is suggested that domestic dogs are endowed with special skills for understanding human communicative behavioural cues [1]. This viewpoint stems from the impressive performance of dogs in the object-choice task that assesses the ability to use human communicative cues to locate a reward hidden in one of two containers. Dogs reliably find the reward by using a range of human communicative cues, such as pointing and head gaze [2]. By contrast, many other animal taxa, such as primates, generally have difficulty finding the reward even when relatively simple human cues are used [2].

One explanation for the impressive skills of dogs in the object-choice task is that human domestication of the dog has yielded genetic changes that give dogs the ability to understand the gestures of their human owners [1, 3]. Evidence given in favour of this is that dogs’ progenitors, the wolf (*Canis lupus*), often perform poorly in object choice tasks [1, 4] and domesticated silver fox puppies (*Vulpes vulpes*) that were experimentally domesticated over several generations are as skilful as dog puppies in the task and perform better than their undomesticated conspecifics [5]. An alternative theory to the domestication hypothesis is that the ability to follow human communicative gestures develops when dogs become human companions and then, over time, they learn how to correctly respond to such gestures during their extensive exposure to them. Evidence for this two-stage hypothesis is that wolves who were raised with extensive socialisation with humans outperformed pet dogs in correctly following human-given cues in the object-choice task [6]. In the same study Udell et al. [6] also found that unlike pet dogs, shelter dogs were unable to use a distal pointing cue to find a hidden reward. Although these findings challenge the domestication hypothesis, Hare et al. [3] criticised Udell et al.’s study [6] for its use of an unorthodox object-choice task. In the standard object-choice task, subjects usually receive warm-up trials that involve the experimenter placing a reward under one of two containers whilst the subject watches. The subject is then allowed to earn the reward by choosing the container that the reward has been placed under. In the warm-up trials of Udell et al.’s [6] study, however, the reward was placed on top of the container so that it was always visible throughout the warm-up trials. Also, in the test trials the food was not hidden inside one of the two containers, which is the normal procedure in the standard object-choice task. Instead the experimenter pointed to one container and if the subject chose correctly the experimenter dropped the reward onto the chosen container. Finally, if a subject failed to make a choice during experimental trials this was coded as an incorrect choice whereas the typical method is to repeat the trial until the subject makes a choice. Hare et al. [3] suggest that using such unorthodox methodology may have resulted in Udell et al.’s [6] negative finding that shelter dogs failed to use a human pointing cue. Hare et al. [3] reported that they had addressed such methodological problems by testing shelter dogs with a standard object-choice task and found that shelter dogs were able to use a human’s point + head gaze cue to locate the hidden reward significantly above chance. Although this suggests unorthodox methodology played a crucial role in the negative findings of shelter dogs ability to use human cues in Udell et al.’s study [6], Udell et al 2010 [7] highlighted that Hare et al. [2] did not test shelter dogs with the same cue that was used by Udell et al. [6] which was a distal momentary point + head gaze cue. When this cue is given the end of the experimenter’s pointing finger is typically more than 50 cm away from the container. In addition, the cue is given before the subject is allowed to make a choice and therefore the cue is absent when the subject is allowed to respond to it. Hare et al. [3] in contrast, used a continuous proximal communicative cue. When this cue is given the end of the experimenter’s pointing finger is only around 15 cm away from the container and the cue remains in place until the subject selects one container. It is therefore likely that a continuous proximal cue is a far easier cue to use than a distal momentary cue. Moreover, because proximal cues are presented so close to the containers there is a risk that subjects choose correctly because of stimulus enhancement rather than understanding the cue’s communicative nature [8, 9].

What is therefore needed to better assess the ability of shelter dogs to use human communicative cues is a study that tests them with distal cues in a standard object-choice task. Here we report the findings of such a study in which we tested shelter dogs with a standard object-choice task and a variety of human communicative cues that included a momentary distal pointing cue that was used by Udell et al. [6] and a proximal pointing cue that was used by Hare et al. [3].

### Experiment 1: Can shelter dogs use a human’s communicative momentary distal point + head gaze cue?

#### Subjects

We tested 20 domestic shelter dogs housed at the University of Queensland Veterinary School’s Clinical Studies Centre, Gatton Campus. The dogs had lived at the shelter for at least one month before they participated in the current study.

The dogs were unwanted/abandoned pets and the centre ran an adoption program that helped rehome the dogs with suitable owners. Only dogs that had passed a behavioural assessment were selected for the adoption program (see supporting material S1 **Form**). The main reason for the behavioural assessment is that whilst housed at the centre the dogs were used to help students learn about animal handling, animal behaviour and veterinary procedures, such as shaving the dog’s fur in preparation for an injection. Therefore, dogs that were aggressive or too timid, for example, would not be suitable for the adoption program.

All dogs housed at the centre received daily interaction with each other in an outside lawned enclosure that had several enrichment items, such as small water baths and pet toys. In addition, volunteer students took the dogs for daily walks around the university campus. The shelter dogs were experimentally naïve and their estimated age ranged from 8 months to 8 years.

#### Ethical statement

The current study was approved by the University of Queensland’s ethics committee (reference 12-PSYCH-4-11-JS) and we conducted the study following the animal welfare laws of Australia. During the study, subjects had access to water ad libitum and they were free to stop participating at anytime during the study.

#### Methods

All experiments were conducted in the same room (3.25 x 4 m) that was familiar to the subjects. For two consecutive days, each subject was brought into the room for 20 minutes by the experimenters who were unfamiliar to the dogs. This allowed the dogs to habituate to the experimenters and experimental set-up. By the end of the habituation phase, 19 subjects seemed very comfortable being in the experimental room with the experimenters. One subject exhibited neophobia towards the containers and was therefore excluded from the study.

#### Warm-up trials

Before experimental trials, subjects received warm-up trials to assess motivation to participate in the general experimental procedure. Warm-up trials involved the experimenter (first author) sitting on the floor behind two opaque containers (Formica plastic cups (10 x 7.5cm)) that were aligned next to each other. To control for olfactory cues, the inside of both containers had been rubbed with the reward that was used throughout the present study (a chicken and veal-flavoured dog biscuit). The handler (second author) walked the subject by its leash and positioned the subject 1.5 metres away directly facing the containers and experimenter. The handler then removed the leash and restrained the subject by gently holding the subject’s collar with one finger. The experimenter called the subject’s name and lifted both containers simultaneously to show the subject they were empty. The experimenter then replaced the containers facedown and simultaneously slid them in opposite directions until they were 120 cm apart. The experimenter called the subject’s name again and held up a reward for the subject to look at. Then from the sitting position the experimenter leaned forward and placed the reward underneath one of the containers and then adopted a neutral position by returning to her normal sitting position, placing her hands on her lap and staring straight ahead. Immediately after adopting this neutral position, the handler released the subject. The experimenter maintained her neutral position until the subject made a choice. A correct choice was scored when the subject touched the baited container first (the container with the reward inside). An incorrect choice was scored if subjects touched the empty container first. If subjects chose correctly, the experimenter lifted the container to reveal the reward and said “good boy” or “good girl” and then gave the reward to the subject. Sometimes subjects retrieved the reward before the experimenter had a chance to give it to them. This happened when subjects knocked the container over and they quickly retrieved the reward or when they quickly retrieved the reward as soon as the container was lifted. Once the subject retrieved the reward, the experimenter lifted the empty container to show subjects that there was no food underneath.

When an incorrect choice was made, the experimenter lifted the container and showed the subject it was empty whilst saying “oops, not in there”, and as the handler put the leash back on the subject, the experimenter lifted the baited container to reveal the reward which she picked up and held it in her hands without giving it to the subject.

A trial was deemed “no-choice” if subjects failed to make contact with a container within 25 seconds. To proceed onto the experimental test phase, subjects were required to make four consecutive correct choices (two for each side) within one session. Each session had a maximum of 12 warm-up trials. Trials were counterbalanced in that the baited container never appeared on the left or right side on more than three consecutive occasions. If subjects failed the first session, they were given a second session on another day. Subjects were excluded from the study if they failed to make four consecutive correct choices within two sessions.

Only 11 of the 19 subjects were able to pass the warm-up trials within two sessions and therefore 8 subjects were excluded from the rest of the study. Four of these excluded subjects were unable to pass the warm-up trials because they consistently failed to choose a container. The 4 other excluded subjects made a choice on each warm-up trial, but they could not reach the criterion of choosing the baited container on four consecutive trials.

**Table 1:** The Subjects’ ages, sex, breed and experimental Participation.

Name	Estimated Age (years)		Breed	Experiments completed
Alice	0.8	F	Pointer cross Dane	1, 2, 3, 4
Decacao	1.5	F	Labrador	1, 2, 3, 4
Delilah	8	F	Greyhound	1, 2, 3, 4
Dougy	2.5	M	Bull Mastiff Dane Cross	1, 2, 3, 4
Lily	2	F	Mastiff Cross	1, 2, 3, 4
Tassie	0.8	M	Pointer Dane Cross	1, 2, 3, adopted
Daisy	2.5	F	Stumpy Tail Cattle cross	1, 2, 3, adopted
Cheeky	1	M	Cavalier poodle Cross	1 Excluded-failed warm-up
Plank	2	M	Neo Mastiff cross	1 Excluded-failed warm-up
Ruby	1	F	Kelpie	1 Excluded-failed warm-up
Sydney	1	F	Cattle Cross	1 Excluded-failed warm-up
Aussie	2	F	Cattle Cross	Excluded- failed warm-up
Bud	0.9	M	Doberman	Excluded- failed warm-up
Darwin	0.8	M	Pointer cross Dane	Excluded- failed warm-up
Dougall	2	M	Bull Arab Cross	Excluded- failed warm-up
Forest	0.75	M	Cattle Cross	Excluded- failed warm-up
Knock	4	M	Greyhound	Excluded- failed warm-up
Petal	1	F	Kelpie Cross	Excluded- failed warm-up
Slinky	2	F	Staffordshire Cross	Excluded- failed warm-up
Shipton	2	M	Staffordshire Cross	Excluded neophobia towards containers

### Experimental trials

#### Subjects

We tested 11 dogs that passed the warm-up trials (see Table 1).

#### Methods

The methods for experimental trials were identical to the warm-up trials apart from the following modifications. When the containers were in front of the experimenter she stood an opaque occluder (55 x 33 cm) in front of them that blocked the subject’s view of the containers. The experimenter then held a reward above the occluder and called the subject’s name until the subject looked at the reward. The reward was then placed on the floor between the containers that were behind the occlcuder and out of the subject’s view. The experimenter then baited one container by lifting it up and placing it over the reward and sham baited the other container by just lifting it and placing it back down again. The container to the experimenter’s right was always baited or sham baited first. The experimenter then removed the occluder and positioned the containers 120 cm apart (as in the warm-up trials). The experimenter then provided the subject with a momentary distal point + head gaze cue to indicate which of the two containers was baited. The cue involved the experimenter looking at the subject then turning her head to look at the baited container whilst simultaneously pointing across her body with her index finger pointing towards the baited container. The distance between the end of the experimenter’s index finger and baited container was approximately 55 cm. The experimenter gave the cue four times before adopting the neutral position that prompted the handler to release the subject to make its choice. If the subject touched one container, the experimenter followed the same procedure as in the warm-up trials. If subjects failed to make a choice within 25 seconds they were allowed to repeat the trial after the first session of test trials.

Seven subjects received two sessions consisting of 8 trials on separate days. Four subjects received the same number of trials but were given two sessions on the same day (with a 15 minute break between each session). These four subjects enrolled onto the pet adoption program midway through the present study and there was not enough time to test them with one session per day.

#### Data scoring and analysis

In all experiments of this study, the handler scored the subjects’ choices live and all trials were video recorded. We used a one-sample t-tests to analyse if subjects were able to use the experimenter’s cue to locate the hidden reward significantly above chance levels. We used the binominal test to analyse whether each subject performed above chance levels (50%) for each cue type.

To assess interobserver reliability, the experimenter assessed the subjects’ performance in 100% of the video trials. In all warm-up and experimental trials of this study, interobserver reliability was excellent with 100% agreement.

#### Results

We found that subjects failed to use the experimenter’s momentary distal point + head gaze cue to locate the hidden reward significantly above chance levels (one-sample t-test: *M =* 7.27, *SD* = 1.85, *t*(10) = -1.31, *p* = .221). Individual analysis revealed that no subject used the momentary or distal cue to locate the food reward significantly above chance (binominal test: *p >* .05). We repeated 9% of the trials because of subjects failing to make a choice on each trial during testing.

#### Discussion

A surprising finding of the warm-up trials is that 8/19 subjects failed to select the baited container on four consecutive trials. Five of these subjects selected a container on each trial and were therefore clearly motivated to participate in the task.

The 11 subjects who passed the warm-up trials were tested for their ability to use an experimenter’s momentary distal cue to locate a hidden reward. We found that shelter dogs failed to use this cue to locate the hidden reward above chance levels. The momentary distal cue is a relatively difficult one for subjects to follow because the cue is not in place when the subject is released to make a choice. In the next experiment we made the cue easier by providing subjects with a distal cue that remained in place when subjects made their choice.

### Experiment 2: Can shelter dogs use a human’s communicative continuous distal point + head gaze cue?

#### Subjects

We tested 11 subjects that had received the momentary distal cue in experiment 1 (see Table 1).

#### Warm-up trials

Subjects received warm-up trials before each experimental session with the same method and procedure that they had received in experiment 1.

Four subjects failed to reach criterion of choosing 4 consecutive baited containers within two sessions and were therefore excluded from experimental test trials (see Table 1).

#### Experimental trials

We tested the 7 subjects that passed the warm-up trials with a similar procedure and method as in experiment 1. The only differences were that subjects received four test sessions consisting of two different human communicative cues. We tested subjects with a continuous distal point + head gaze cue. This cue only differed from the momentary distal point + head gaze cue in that it remained in place during the time subjects were released to make their choice i.e., at the time of choice the experimenter was continuously pointing and looking at the baited container. Subjects were also tested with the same momentary distal point + head gaze cue that they had received in experiment 1. This allowed us to compare the subjects’ performance on this cue with the continuous distal point + head gaze cue.

Subjects received 16 trials of each cue over 4 sessions. Each session consisted of 4 momentary distal point + head gaze cues and 4 continuous distal point + head gaze cues. The cues were presented pseudo-randomly and counterbalanced for left and right with no cue being presented on the same side on more than three consecutive occasions.

#### Results

Dogs failed to use either of the experimenter’s distal cues to locate the reward significantly above chance (one sample t-tests: momentary, *M =* 7.14, *SD* = 1.95, *t*(6) = -1.16, *p* = .289; continuous, *M =* 8.71, *SD* 1.38, *t*(6) = 1.37, *p* = .220). Individual analysis revealed that no subject used the momentary cue or distal cue to locate the reward significantly above chance (binominal test: *p >* .05). Less than 4% of the trials were repeated because of subjects failing to make a choice during testing.

#### Discussion

As in experiment 1, we found that not all subjects could pass the warm-up trials as four subjects failed to find the baited reward within two sessions. These subjects made a choice on each trial and were therefore clearly motivated to participate in the task. Interestingly, these subjects passed the warm-up trials in experiment 1 and there could be several reasons why they failed the warm-up trials in the experiment 2. For example, it is possible that they were less attentive to the trials or were more distracted that affected recalling which container the food was hidden underneath.

In experimental trials, we found that shelter dogs failed to use a human’s continuous distal point + head gaze cue to locate the reward. And, as in experiment 1, they failed to use a momentary distal point + head gaze cue. In contrast, many studies have shown that pet dogs are able to follow both of these cues [2]. It therefore appears that, unlike pet dogs, shelter dogs lack the ability to spontaneously use human momentary or continuous distal cues to locate a hidden reward.

In past object-choice studies that have tested the abilities of dogs to use human communicative cues the standard procedure is that the human experimenter does not vocalise when giving the cue, such as calling the subject’s name [2]. It is possible that when providing communicative cues in such studies, vocalisations are not important for pet dogs because of their previous extensive social interaction with humans. But shelter dogs are likely to have limited human social interaction. Vocalisations, therefore, may be more important for shelter dogs because, for example, they are less tuned in to human behaviour than pet dogs.

In the next experiment we investigated if shelter dogs could use human communicative distal cues when they were given with vocalisations.

### Experiment 3: Can shelter dogs use human vocalised communicative distal cues?

#### Subjects

We tested the 7 subjects that completed experiment 2 (see Table 1). All subjects passed the warm-trials before each test session.

#### Methods

We tested subjects with the non-vocal momentary cue and the non-vocal distal cue that we used in experiment 2. In addition, we tested subjects with these two cues but the experimenter vocalised when giving the cue. Vocalised cues involved the experimenter calling the subject’s name each time she provided the cue, which was a total of four times for each trial. Apart from the vocalisations, the experiment was conducted in the same way as in experiment 2. Subjects received one test session per day that consisted of 8 trials (each of the four cues presented two times). In total subjects received 8 sessions and 16 trials for each of the four different cues.

#### Results

Shelter dogs located the hidden reward significantly above chance when the experimenter provided a vocalised continuous distal cue, (one-sample t-test: *M =* 10.57, *SD =* 2.15, *t*(6) = 3.17, *p* = .019) but they did not locate the reward significantly above chance with any other cue: (one-sample t-test: vocalised momentary distal, *M =* 8.57, *SD =* 2.23, *t*(6) = 0.68, *p* = .522; momentary distal, *M = 9*.*14*, *SD =* 2.41, *t*(6) = 1.26, *p* = .256, and continuous distal, *M =* 9.14, *SD =* 1.68, *t*(6) = 1.80, *p =* .121). When directly comparing the subjects’ performance in the vocalised and non-vocalised continuous distal cues there was only a marginal significant difference between the two cues (paired t-test: *M* = -1.43, *SD* = 1.62, *t*(6) = -2.34, *p* = 0.058).

Individual analysis revealed that only two subjects (Decacao and Dougy) used the vocalised momentary distal cue to locate the reward significantly above chance with both subjects scoring 13/16 correct (*p* = .021). Decacao was the only subject to perform significantly above chance with the momentary distal cue scoring 13/16 correct (*p* = .021). No subject performed significantly above chance levels when tested with the vocalised momentary distal cue or the continuous distal cue (*p >* .05). No trials were repeated as subjects made a choice in every trial.

#### Discussion

When tested with continuous distal cues we found that vocalisations helped shelter dogs to use a human’s communicative cue to locate a hidden reward. Subjects found the reward significantly above chance levels when the cue was presented with vocalisations but not when the cue was presented without vocalisations. However, directly comparing the subjects’ performance in the vocalised and non-vocalised continuous distal cues only shows a marginal significant difference between the two cues. It should be noted, however, that we could only test a small sample size (7 subjects) with these cues and therefore testing a larger sample size might reveal a more significant effect.

In contrast to the continuous cue, we found vocalisations made no difference to the subjects’ performance when tested with a momentary distal cue as they failed to locate the reward significantly above chance levels in both vocalised and non-vocalised versions of the cue.

As we discussed earlier, momentary cues are more difficult than continuous cues because the experimenter stops giving the cue just before subjects are allowed to approach the containers to make a choice. And distal cues are likely to be more difficult than proximal cues because proximal cues may cause a stimulus enhancement effect. The only human social cue that Hare at el. [3] tested with shelter dogs was a continuous proximal cue. We delayed testing shelter dogs with this cue. If we had tested subjects with the proximal cues before, or in tandem, of distal cues then any possible stimulus enhancement from the use of proximal cues could have helped subjects learn about distal cues. However, now that shelter dogs had been exclusively tested with distal cues, we wanted to investigate if they could use the same continuous proximal cue that was used by Hare et al. [3]. In addition, we tested subjects with a momentary proximal cue to investigate if subjects had the ability to use an easier momentary cue compared to the momentary distal cue.

### Experiment 4: Can shelter dogs use a human’s communicative proximal point + head gaze cue?

#### Subjects

We tested 5 of the 7 subjects that we tested in experiment 3. Two subjects from experiment 3 were adopted and therefore unavailable for further testing (see Table 1).

#### Method

The set-up and procedure only differed from experiment 3 by the following two modifications. After hiding the food, the experimenter placed the containers 100 cm apart, instead of 120 cm, and when she pointed at the baited container her arm was more extended than in the previous experiment. These two modifications were necessary to ensure that the end of the experimenter’s index finger was approximately 10–15 cm from the baited container therefore qualifying as a proximal cue.

We presented subjects with non-vocalised cues and each subject received 16 trials of each cue over 4 sessions. Each session consisted of 4 momentary proximal cues and 4 continuous proximal cues that were presented pseudorandomly and counterbalanced for side as in the previous experiments. All subjects passed the warm-up trials prior to the test sessions.

#### Results

Shelter dogs significantly located the hidden reward above chance levels for both types of communicative cue (one-sample t-test: momentary proximal cue, *M =* 13.8, *SD =* 2.39, *t*(4) = 5.43, *p* = .006; continuous proximal cue, *M =* 15.2, *SD =* .84, *t*(4) = 19.24, *p* < .001). Individual analysis revealed that all subjects performed significantly above chance levels when tested with the continuous proximal cue, with Delilah and Decacao scoring 16/16 (*p* < .001), Alice and Lily 15/16 (*p* = .001) and Dougy, 14 /16 (*p* = .004). When tested with momentary proximal cues, four subjects scored significantly above chance levels (Decacao 16/16 correct (*p* < .001), Delilah and Dougy 15 /16 (*p* = .001) and Lily 13 /16 (*p* = .021)). No trials were repeated as subjects made a choice in every trial.

#### Discussion

We found that shelter dogs could use a human’s continuous proximal cue to locate a hidden reward significantly above chance. This finding is consistent with the results of Hare et al. [3] who also reported that shelter dogs could use the same continuous proximal cue to locate the reward above chance levels. We also found that shelter dogs could use a momentary proximal cue to locate a hidden reward significantly above chance. This was the first time during our study that shelter dogs used a momentary cue to consistently locate the hidden reward. However, in our previous three experiments the momentary cue was always distal. Therefore, this suggests that success in using the proximal momentary cue was based on stimulus enhancement rather than understanding the communicative nature of the cue. Alternatively, because subjects were tested repeatedly they may have learnt across experiments to better utilize the experimenter’s communicative cues.

Finally, it could be argued that the small sample size we tested in experiment 4 could have affected the positive findings. However, we do not think this is the case because at the individual level all subjects in our last experiment located the hidden reward significantly above chance when tested with the continuous proximal cue and 4/5 subjects did so when tested with the momentary proximal cue. These two cues were presented without the experimenter vocalising and if we compare the performance of individuals tested with non-vocalised cues in our previous experiments, in which the sample size was greater than 5, we see that no subject performed significantly above chance in experiment 1 and 2 when tested with distal cues and momentary cues, and only one subject performed significantly above chance in experiment 3 when tested with a momentary distal cue.

## General discussion

We investigated the abilities of shelter dogs to understand human communicative cues in a standard object-choice task that included both proximal and distal cues that were presented continuously or momentary. In experiment 1, we found that shelter dogs were unable to use a momentary distal pointing + head gaze cue to locate the hidden reward above chance levels. In experiment 2, we used an easier version of the distal cue by presenting it continuously instead of momentary. Again, we found shelter dogs were unable to use the cue to locate the reward above chance levels. Many published studies have shown that pet dogs are able to use both these cues to locate a hidden reward in object-choice tasks [2]. However, compared to shelter dogs, pet dogs are likely to have much more interaction with humans. It is possible, then, that this experience helps pet dogs to develop their abilities to follow a range of human communicative cues that differ in their level of difficulty. During this development, human vocalisations may play an important role in subjects learning to use human communicative cues. It is difficult to envisage owners giving non-vocalised communicative cues during their daily interaction with their pet dogs. It is probable that there is constant vocalised feedback from the owners when giving communicative cues. For instance, if an owner throws a ball and their dog fails to see where it landed, then the owner could keep pointing to the location whilst simultaneously calling the dog’s name until the dog finally finds it. Such vocalisations may, for example, help the dog to tune into the cue more effectively or re-attend to the cue if it is distracted. Once dogs have extensive experience of such interactions it is likely to be easier for them to make the transition to using non-vocalised human communicative cues.

In past studies that tested pet dogs’ understanding of human communicative cues it is typical that the experimenter did not vocalise when giving the cue [2]. However, a non-vocalised distal cue may be too difficult for shelter dogs who are likely to have had much less human social interaction than pet dogs. In experiment 3, we tested shelter dogs with a vocalised and non-vocalised version of the distal momentary cue and the distal continuous cue that we had used in experiment 1 and 2.

We found shelter dogs were able to use a vocalised distal continuous cue to reliably locate the reward above chance levels, but they failed to do so with the vocalised distal momentary cue. And again, as in experiment 1 and 2, shelter dogs were unable to use a distal non-vocalised momentary cue and a non-vocalised distal continuous cue to locate the reward above chance.

When directly comparing the subjects’ performance in the vocalised and non-vocalised continuous distal cues, we found only found marginal significant difference. This could be owing to the small sample size of 7 subjects that we tested and therefore future research with a larger sample size is needed to test the effect of vocalisations on the performance of shelter dogs in the object-choice task. Interestingly, and to the best of our knowledge, there is no study that has tested dogs with a vocalised distal continuous point + head cue in the standard object choice task [2]. However, similar experimental paradigms have shown that communicative cues + verbal cues facilitate performance compared to when each cue is given independently. For instance, pet dogs were quicker to respond to their owners’ request to retrieve an item when a gestural + verbal cue were used compared to when only a verbal or a gestural cue was used [10]. And a verbal cue of communicative intent given simultaneously with a communicative gesture [11] or immediately preceding the gesture [12] was beneficial to dogs in gaze-following tasks compared to when the verbal cue of intent is absent.

Interestingly, vocalisations also seem to help great apes follow human cues in the object choice task, e.g., orangutans and chimpanzees, [13]. And similarly to shelter dogs, these great apes were not exposed to extensive human social interactions that pet dogs usually receive.

In our final experiment we found that shelter dogs could reliably locate the hidden reward with a continuous proximal point + head gaze cue. This is the only human communicative social cue that Hare et al. [3] used in their study and they also found shelter dogs were successful at using this cue. However, as we discussed earlier, proximal cues may involve stimulus enhancement in that the subject’s attention is naturally drawn to the correct container because the movement of the hand is so close to it during cuing. In support of this is that in our final experiment shelter dogs were successful at using a non-vocalised momentary proximal cue. But the same subjects never showed any success in using the non-vocalised distal momentary cue in which the cue was presented farther away from the container and therefore excluded the possibility of stimulus enhancement.

Finally, a surprising finding of our study is that we excluded many subjects because they failed the warm-up trials. In total, 12 subjects failed to select the baited container on four consecutive warm-up trials within 24 trials. And 9 of these subjects always made a choice in every trial so they were clearly motivated to participate in the task. It could be argued that the shelter dogs we tested had problems passing the warm-up trials owing to temperamental issues that were a consequence of their rearing history. However, we think this is unlikely. All of the shelter dogs we tested had passed a behavioural assessment for negative temperamental traits such as aggression and timidness.

The finding that many of our subjects failed the warm-up trials within 24 trials is even more perplexing considering that Hare et al. [3] reported that all of the 23 shelter dogs they tested passed the warm-up trials within 8 trials. One possible explanation for this difference is the method we used to hold and release the dogs (see S1 and S2 videos), which was different to the one used by Hare et al. [3] When the experimenter gave the communicative cue in our study, the handler held the subjects by their collar and then released them to make their choice by simply letting go of the collar. In contrast, the handler in Hare et al.’s [3] study continuously held the subject with an unusually long leash throughout each trial. This meant that the handler not only held the dog by the leash when it approached the containers, but also when the subject chose a container. However, continually holding a dog by a leash during warm-up, or experimental trials may, for example, cause the handler to inadvertently influence the dog’s behaviour during trials.

It would be interesting to know if the handlers in other object-choice task studies held dogs continuously by the leash throughout warm-up trials and test trials. Unfortunately, most published studies fail to report the exact method of subject restraint and release. In the 22 object-choice task dog studies reviewed by Mulcahy & Hedge [2] only four studies explicitly reported the exact method. In three studies the handler held the subject by the collar and then released the subject [14–16] and in one study the handler restrained the subjects by the leash and released them by letting go of the leash [4].

Methodological differences are a serious problem in object-choice task studies [2] and this is exacerbated by many published studies containing methodology that is often inadequate for faithful replication. This problem can easily be resolved by authors publishing video samples of warm-up and experimental trials (e.g., [17]) but these are rarely included in published object-choice studies.

In conclusion, domesticated shelter dogs consistently failed to use the range of non-vocalised distal cues in the object-choice task. Therefore, our findings suggest ontogenetic factors, such as high levels of social interaction between domesticated dogs and humans, play an important role in the development of dogs’ understanding of human communicative cues. The domestication process may have resulted in genetic traits that facilitate this development, such as a predisposition to attend to humans [17] but our findings fail to support that domesticated dogs have evolved specialised skills to understand human communicative cues during and as a result of human domestication.

To the best of our knowledge, our study is the first to test shelter dogs with a standard object-choice task and with a range of momentary and continuous cues. Yet there are numerous studies that have tested pet dogs this way. More research with shelter dogs is therefore needed to address this disparity. But researchers should ensure that they use the standard object-choice method with the range of cues that pet dogs normally receive. This will help to better assess the abilities of shelter and pet dogs in their understanding of human communicative behaviour.

## Supporting information

S1 Form Assessment form. Behavioural Assessment used by the Clinical Studies Centre.
(PDF)

S1 Table Experiment 1: Can shelter dogs use a human’s communicative momentary distal point + head gaze cue? (PDF)

S2 Table Experiment 2: Can shelter dogs use a human’s communicative continuous distal point + head gaze cue? (PDF)

S3 Table Experiment 3: Can shelter dogs use human vocalised communicative distal cues? (PDF)

S4 Table Experiment 4: Can shelter dogs use a human’s communicative proximal point + head gaze cue? (PDF)

S1 Video Example of a warm-up trial. (AVI)

S2 Video Example of an experimental trial (Experiment 4 continuous proximal cue). (AVI)
