# Supplementary material for: Reassessing shelter dogs’ use of human communicative cues in the standard object-choice task
Source: PLoS One. 2019 Mar 7;14(3):e0213166. doi: 10.1371/journal.pone.0213166 (PMC6405081; doi:10.1371/journal.pone.0213166)
Supplement: S1 Form — Behavioural Assessment used by the Clinical Studies Centre. (PDF) [file pone.0213166.s001.pdf]

S1 Canine Behaviour Assessment Form

**Name:** ____________<br><br>**Breed:** ____________	**Assessor:** ___________<br><br>**Observer:** __________

General background information if known:
Date in:
Origin:
Known history:

Initial Observations

On arrival to the kennel to get the dog, is the dog:

Happy to see you	☐	Calm	☐	Confident	☐
Anxious/cautious	☐	Disinterested	☐	Backing away	☐
Submissive/Timid	☐	Frustrated	☐	Destructive	☐
Lunging at bars	☐	Barking	☐	Growling/snarling	☐

Describe:

General Character

Social	☐	Cautious	☐	Over-excited	☐	Unfriendly	☐

Describe:

Basic Commands

1. Sit yes/no
2. Down yes/no
3. Stay yes/no
4. Come yes/no
5. Other:

Toy Interaction

Increasing value of: **1** **2** **3**

No interest ☐ Interactive ☐ Difficulty retrieving/hoards ☐

Describe:

Food Refusal

Increasing value of: **1** **2** **3**

Focused ☐ Displacing ☐ Demanding ☐ Uninterested ☐

Describe:

Play Preference

Uninterested ☐ Chase games ☐ Plays alone ☐

Retrieve & Relinquish ☐

Describe:

<u>Handling</u>

1. <u>Stroking</u>: (along back)

Seeks affection	☐	Over excited	☐	Remained Still	☐
Became mouthy	☐	Growl/snarl/snap	☐ **(STOP Assessment)**		

Describe:

2. <u>Head pats</u>:

Seeks affection	☐	Over excited	☐	Remained Still	☐
Became mouthy	☐	Growl/snarl/snap	☐ **(STOP Assessment)**		

Describe:

3. <u>Muzzle tolerance</u>:

Seeks affection	☐	Over excited	☐	Remained Still	☐
Tolerant	☐	Mouthy	☐	Difficult to examine	☐
Growl/snarl/snap	☐ **(STOP Assessment)**				

Describe:

4. <u>Touch tolerance</u>: (ears, feet, tail, and body)

Seeks affection	☐	Over excited	☐	Remained Still	☐
Tolerant	☐	Became mouthy	☐		
Growl/snarl/snap	☐ **(STOP Assessment)**				

Describe:

Restraint

Comfortable ☐ Froze ☐ Struggles ☐ Mouths ☐

Growl/snarl/snap ☐ **(STOP Assessment)**

Describe:

Food/Feeding

Comfortable ☐ Froze ☐ Ate fast ☐ Growls ☐

Snapped ☐

Describe:

Dog – to - Dog

1. Breed: ………………. Gender: M ☐ / F ☐ Desexed: Y ☐ / N ☐

Politely social ☐ Play ☐ Barks ☐ Unsure/avoids interaction ☐

Pushy/rough ☐ Lunges forward/growls/snarls ☐ Fight ☐

Describe:

2. Breed: ………………. Gender: M ☐ / F ☐ Desexed: Y ☐ / N ☐

Politely social ☐ Play ☐ Barks ☐ Unsure/avoids interaction ☐

Pushy/rough ☐ Lunges forward/growls/snarls ☐ Fight ☐

Describe:

Other tests required?

1. ________________ Describe:

2. ________________ Describe:

3. ________________ Describe

Passed		Kennel Program:<br>Basic ☐<br>Individualised ☐	Signed:<br>1.<br>2.
Failed		Reason:	

NOTE: The above information is based on observation of the animal at the time of assessment and any history that may have been supplied. A temperament assessment is never perfect –it relates to the context the animal is in at the time and any individual animal may behave differently in different contexts. The University of Queensland and its employees and agents accept no responsibility in the event that the animal behaves differently nor are any guarantees made in respect to their future behaviours.

RECOMMENDATIONS

1. **Fences:**
   a) 4' secure to the ground ☐
   b) 6' secure to the ground ☐
   c) Solid fence ☐ Height: _____
2. **Guardian:**
   a) Inexperienced adopter/s ☐
   b) Experienced adopter/s ☐
   c) Suitable for a fit older person ☐
   d) Suitable for relatively frail person ☐
   e) Suitable for children ☐ Age: ______

f) Not suitable for older/ relatively frail person ☐

g) Not suitable for children ☐

3. Home Life:

a) Dog requires human company for most of the day ☐

b) Dog may cope for up to 6 hours alone (with enrichment) ☐

4. Other Dogs:

a) Not to live with other dogs ☐

b) Must live with other dog/s ☐

c) Would prefer to live without other dogs ☐

d) Can live with or without other dogs ☐

e) Can live with or without other dogs BUT recommend ongoing socialisation ☐

f) Recommend home with other dogs ☐

5. Training:

a) Formal Obedience ☐ c) Socialisation ☐

b) Agility ☐ d) Other ☐

END
