# Supplementary material for: Reassessing shelter dogs’ use of human communicative cues in the standard object-choice task
Source: PLoS One. 2019 Mar 7;14(3):e0213166. doi: 10.1371/journal.pone.0213166 (PMC6405081; doi:10.1371/journal.pone.0213166)
Supplement: S1 Table — (PDF) [file pone.0213166.s002.pdf]

Experiment 1: Can shelter dogs use a human's communicative momentary distal point + head gaze cue?

**Trial**	Alice	Aussie	Cheeky	Daisy	Decacao	Delilah	Dougy	Lily	Plank	Ruby	Sydney	Tassie
**1**	0	1	1	1	1	0	1	0	1	0	1	1
**2**	0	0	0	1	0	0	0	0	0	1	1	1
**3**	1	0	1	1	1	0	1	1	1	0	1	1
**4**	0	0	0	0	0	1	1	0	0	1	0	0
**5**	0	0	1	1	1	1	0	0	1	0	1	0
**6**	0	0	0	0	1	0	1	0	0	0	1	1
**7**	0	1	1	0	1	0	1	1	0	0	0	1
**8**	1	1	0	1	0	0	0	1	1	0	0	0
**9**	0	-	1	1	1	0	1	0	1	0	1	1
**10**	0	-	0	0	0	0	0	0	0	1	1	0
**11**	0	-	1	0	0	0	1	0	0	0	0	1
**12**	0	-	1	0	0	1	0	0	1	0	0	0
**13**	1	-	1	0	0	1	0	1	1	0	1	1
**14**	0	-	0	0	0	0	1	1	0	0	0	1
**15**	1	-	1	0	0	0	1	1	1	1	1	1
**16**	1	-	0	1	0	1	0	1	0	1	0	0
**Total correct**	**5**	***Excluded***	**9**	**7**	**6**	**5**	**9**	**7**	**8**	**5**	**9**	**10**
