# Supplementary material for: Reassessing shelter dogs’ use of human communicative cues in the standard object-choice task
Source: PLoS One. 2019 Mar 7;14(3):e0213166. doi: 10.1371/journal.pone.0213166 (PMC6405081; doi:10.1371/journal.pone.0213166)
Supplement: S2 Table — (PDF) [file pone.0213166.s003.pdf]

Experiment 2: Can shelter dogs use a human's communicative continuous distal point + head gaze cue?

Mom = Momentary Distal Cue Cont = Continuous Distal Cue

**Trial**	Alice		Daisy		Decacao		Delilha		Dougy		Lily		Tassie	
	Mom	Cont	Mom	Cont	Mom	Cont	Mom	Cont	Mom	Cont	Mom	Cont	Mom	Cont
**1**	0	1	1	1	1	0	0	1	1	0	1	0	0	0
**2**	0	0	1	1	0	1	0	0	0	1	0	1	0	0
**3**	0	1	0	0	0	0	0	1	1	0	1	0	0	1
**4**	0	1	1	1	0	0	1	0	0	1	0	1	0	1
**5**	0	0	1	1	1	1	1	0	0	1	1	1	0	1
**6**	0	1	1	1	1	0	0	1	1	0	0	0	0	0
**7**	1	0	1	1	0	0	1	0	0	1	1	0	1	1
**8**	0	1	0	1	0	0	0	1	1	0	0	1	1	1
**9**	1	1	1	0	1	1	1	1	1	0	1	1	0	0
**10**	1	0	1	1	1	1	0	1	0	1	1	1	0	0
**11**	0	1	0	0	0	0	0	1	0	1	1	0	1	1
**12**	0	1	1	0	0	0	0	1	1	0	0	0	1	0
**13**	1	0	0	1	0	1	0	0	0	1	1	0	0	1
**14**	0	0	1	0	1	0	1	0	1	0	1	0	1	1
**15**	0	1	0	1	1	1	0	1	1	0	0	1	1	0
**16**	1	1	0	0	0	1	0	1	0	1	0	0	0	1
**Total correct**	**5**	**10**	**10**	**10**	**7**	**7**	**5**	**10**	**8**	**8**	**9**	**7**	**6**	**9**
