# Supplementary material for: Reassessing shelter dogs’ use of human communicative cues in the standard object-choice task
Source: PLoS One. 2019 Mar 7;14(3):e0213166. doi: 10.1371/journal.pone.0213166 (PMC6405081; doi:10.1371/journal.pone.0213166)
Supplement: S3 Table — (PDF) [file pone.0213166.s004.pdf]

Experiment 3: Can shelter dogs use human vocalised communicative distal cues?

M = Momentary Non-Vocal Cue VM= Momentary Vocal Cue C= Continuous Non-Vocal Cue VC = Continuous Vocal Cue

**Trial**	Alice				Daisy				Decacao				Delilah				Dougy				Lily				Tassie			
	M	VM	C	VC	M	VM	C	VC	M	VM	C	VC	M	VM	C	VC	M	VM	C	VC	M	VM	C	VC	M	VM	C	VC
**1**	0	1	0	1	1	1	1	0	1	0	1	0	1	0	1	1	1	1	1	1	0	1	1	0	1	0	1	0
**2**	1	0	1	0	1	1	0	1	0	1	1	1	1	1	1	1	1	0	1	0	0	0	1	1	0	0	0	0
**3**	0	0	1	1	1	1	0	1	1	1	0	0	0	1	1	0	0	1	0	1	0	0	0	0	1	1	0	0
**4**	1	1	0	0	0	0	0	1	0	0	1	1	1	0	0	1	0	0	1	0	0	1	1	0	0	0	1	1
**5**	0	1	0	1	1	1	1	0	1	0	1	1	1	0	0	0	1	0	1	1	1	0	0	1	1	0	1	0
**6**	1	0	1	0	0	1	0	1	1	1	0	1	1	1	1	1	0	1	0	1	0	0	1	1	0	1	0	1
**7**	0	0	1	1	1	1	0	1	1	1	1	1	0	1	1	0	1	0	0	1	1	1	0	1	1	1	0	0
**8**	1	1	0	0	1	0	1	1	1	1	1	1	1	1	1	1	0	0	1	1	0	0	0	1	0	0	1	1
**9**	0	0	1	1	1	1	0	0	1	0	1	1	0	1	0	1	1	0	1	1	0	1	0	0	1	1	0	0
**10**	1	1	0	0	0	0	1	1	1	1	1	0	0	1	0	1	0	0	1	1	1	0	1	0	0	0	1	1
**11**	0	1	0	1	1	0	1	0	1	1	1	1	1	0	0	1	1	0	1	1	1	0	0	1	1	0	1	0
**12**	1	0	1	1	0	1	0	1	1	1	0	1	1	1	1	1	0	1	0	1	0	0	0	1	0	1	0	1
**13**	1	1	1	1	1	0	1	0	1	0	1	1	1	0	0	0	1	0	1	0	1	0	1	1	0	0	1	0
**14**	1	1	1	0	0	1	1	1	0	1	0	1	1	1	1	1	0	1	0	1	0	1	0	1	0	1	0	1
**15**	1	1	1	0	1	1	0	1	1	1	1	1	0	1	1	0	1	1	0	1	0	1	0	0	1	1	0	0
**16**	0	0	0	1	0	0	1	1	1	1	1	1	1	1	1	1	0	0	1	1	1	0	1	1	0	0	1	1
**Total correct**	**9**	**9**	**9**	**9**	**10**	**10**	**8**	**11**	**13**	**11**	**12**	**13**	**11**	**11**	**10**	**11**	**8**	**6**	**10**	**13**	**6**	**6**	**7**	**10**	**7**	**7**	**8**	**7**
