# Supplementary material for: Reassessing shelter dogs’ use of human communicative cues in the standard object-choice task
Source: PLoS One. 2019 Mar 7;14(3):e0213166. doi: 10.1371/journal.pone.0213166 (PMC6405081; doi:10.1371/journal.pone.0213166)
Supplement: S4 Table — (PDF) [file pone.0213166.s005.pdf]

Experiment 4: Can shelter dogs use a human's communicative proximal point + head gaze cue?

Mom = Momentary Cue   Con = Continuous Cue

**Trial**	Alice		Decacao		Delilah		Dougy		Lily	
	Mom	Continuous	Momentary	Continuous	Momentary	Continuous	Momentary	Continuous	Momentary	Continuous
**1**	1	1	1	1	1	1	1	0	1	1
**2**	0	1	1	1	1	1	0	1	1	1
**3**	1	1	1	1	1	1	1	0	1	0
**4**	0	0	1	1	1	1	1	1	1	1
**5**	1	1	1	1	1	1	1	1	0	1
**6**	0	1	1	1	1	1	1	1	1	1
**7**	1	1	1	1	1	1	1	1	1	1
**8**	1	1	1	1	1	1	1	1	1	1
**9**	0	1	1	1	1	1	1	1	1	1
**10**	1	1	1	1	1	1	1	1	1	1
**11**	0	1	1	1	1	1	1	1	1	1
**12**	1	1	1	1	0	1	1	1	1	1
**13**	1	1	1	1	1	1	1	1	0	1
**14**	0	1	1	1	1	1	1	1	1	1
**15**	1	1	1	1	1	1	1	1	0	1
**16**	1	1	1	1	1	1	1	1	1	1
**Total correct**	**10**	**15**	**16**	**16**	**15**	**16**	**15**	**14**	**13**	**15**
